# Noise-induced hearing loss among military personnel in Saudi Arabia: a preliminary study

**DOI:** 10.25122/jml-2025-0013

**Published:** 2025-06

**Authors:** Ahmad Alanazi, Abrar AlMutairi, Fulwah AlHargan

**Affiliations:** 1 Department of Audiology and Speech Pathology, College of Applied Medical Sciences, King Saud bin Abdulaziz University for Health Sciences, Riyadh, Saudi Arabia; 2 King Abdullah International Medical Research Center, Riyadh, Saudi Arabia; 3 ENT Division, King Abdulaziz Medical City, Riyadh, Saudi Arabia; 4 Research Unit, College of Applied Medical Sciences, King Saud bin Abdulaziz University for Health Sciences, Riyadh, Saudi Arabia

**Keywords:** awareness, hearing loss, hearing protection, military, noise, Saudi Arabia, tinnitus

## Abstract

Noise-induced hearing loss (NIHL) is a leading cause of sensorineural hearing loss (SNHL), with significant global prevalence. Occupational, environmental, and recreational noise exposure has heightened concerns about NIHL in Saudi Arabia. Despite general awareness of noise-related auditory risks, misconceptions about safe exposure durations and listening practices persist. This cross-sectional preliminary study investigated the effects of noise exposure on military personnel in Riyadh, Saudi Arabia, using a structured approach that included case histories and audiological assessments. The study included 40 male participants, primarily aged 41–50 years, with an extensive military service. Case histories and audiological assessments revealed that 77.5% experienced prolonged occupational noise exposure. Tinnitus was the most common symptom (60%), and bilateral hearing loss was more prevalent (87.5%) than unilateral. Audiometric findings revealed distinct SNHL profiles among the participants, particularly those with prolonged exposure durations. While prolonged exposure correlated with more severe hearing loss, statistical significance was not achieved (*P* = 0.60). The participants with more than 2 years of experience reported tinnitus (61.8%) and dizziness (14.7%). The findings align with global evidence linking military environments to the high prevalence of NIHL due to hazardous noise levels. Bilateral SNHL patterns and the predominance of tinnitus underscore the cumulative auditory damage associated with chronic exposure. The study emphasizes the importance of implementing effective hearing conservation programs, which include regular auditory assessments, mandatory use of hearing protection, and education on the risks of NIHL. Addressing these factors is critical to mitigating the societal and occupational impacts of NIHL in Saudi Arabia.

## INTRODUCTION

Noise-induced hearing loss (NIHL) is a prevalent form of sensorineural hearing loss (SNHL) resulting from prolonged exposure to high levels of noise. Globally, it is the second most common cause of hearing loss after age-related hearing loss, affecting approximately 5% of the population and typically more prevalent in adult men [1]. In Saudi Arabia, the increasing exposure to occupational, environmental, and recreational noise has raised concerns about the incidence of NIHL among its population. Several studies have examined the awareness and prevalence of NIHL in Saudi Arabia. A cross-sectional study conducted in the southern region revealed that while a significant majority (88.5%) of participants acknowledged that high sound levels could adversely affect hearing, only 9.5% correctly identified the minimum duration of exposure that could harm hearing [2]. This knowledge gap underscores the need for enhanced public education on the risks associated with noise exposure. Another study focusing on the Makkah region assessed the relationship between NIHL awareness and the use of personal listening devices (PLDs). The findings indicated a general awareness of NIHL among the population; however, misconceptions persisted regarding safe listening practices and the risks posed by PLDs [3]. This highlights the importance of targeted interventions to promote safe listening habits, especially among younger demographics who are frequent users of PLDs.

The prevalence of NIHL in Saudi Arabia varies across different regions and occupational settings. For instance, a study conducted in the Al Baha region suggested gaps in community knowledge regarding the causes and protective measures against NIHL [4]. Additionally, research among industrial workers in the country reported a prevalence rate of 48%, indicating a substantial occupational risk [5]. These findings underscore the importance of stringent occupational health regulations and the implementation of effective hearing conservation programs. Continuous exposure to high-decibel environments, such as firearms, heavy machinery, and aircraft, renders military members particularly susceptible to auditory impairments. A study focusing on military personnel in Al Ahsa, Saudi Arabia, reported a high prevalence of hearing impairment, with 71.6% of participants exhibiting signs of NIHL. This elevated rate is attributed to prolonged exposure to hazardous noise levels and insufficient use of hearing protection devices [6]. Similarly, research on Saudi military pilots indicated that 18.4% suffered from NIHL, with fixed-wing pilots experiencing a higher prevalence (42%) compared to their rotary-wing counterparts. The study found a significant correlation between total flight hours and the degree of hearing loss, underscoring the cumulative effect of noise exposure over time [7].

These findings align with global data highlighting the vulnerability of military personnel to NIHL. The US Department of Veterans Affairs, for instance, identifies hearing loss and tinnitus as the most common service-connected disabilities among veterans [8]. The military environment often involves exposure to impulse noises exceeding 150 dB, levels at which even brief exposure can cause immediate and irreversible damage to the auditory system [9]. The global burden of NIHL is significant, with substantial physical, mental, social, and economic impacts on individuals and societies. Stress and social isolation resulting from hearing impairment contribute to quality-of-life decrements that often go undetected [1]. In Saudi Arabia, the societal implications of NIHL are compounded by limited awareness and preventive measures, necessitating comprehensive public health strategies to address this growing concern. This study aimed to investigate the impact of noise exposure on hearing among Saudi military personnel and establish preliminary methods prior to determining the need for larger, comprehensive research.

## MATERIAL AND METHODS

This cross-sectional preliminary study aimed to evaluate the effects of noise exposure on hearing among Saudi military personnel, as well as to assess the feasibility, methodology, and potential outcomes of a larger-scale investigation. No identifiable or health information was collected, and access to the data was restricted to the authors. Written informed consent was obtained from all participants before their inclusion in the study. Confidentiality of data was maintained, and participants were informed about their right to withdraw from the study at any time without consequences.

### Participants

Participants included both active and retired military personnel who had been exposed to loud noise during their service and who visited the audiology clinic at King Abdulaziz Medical City in Riyadh, Saudi Arabia, for hearing assessment. Exclusion criteria included individuals not exposed to loud noise, those over 60 years of age, and those with a family history of hearing loss, known hearing disorders (e.g., otitis media, Meniere’s disease), or regular exposure to noisy recreational activities (e.g., firearm use, motorcycle riding).

### Procedure

The study followed a structured approach (i.e., case history and audiological assessment) to ensure comprehensive data collection and reliability of results.

### Case history documentation

Each participant was asked to complete a standardized case history using a specifically designed form to ensure the documentation of crucial information for the study and, equally important, to support the analysis and interpretation of the data (Supplementary file S1). The form collected demographic and clinical data, including gender, age, duration of military service and noise exposure, presence of tinnitus, dizziness, or any other auditory complaints, reports of hereditary hearing loss, and information on other medical conditions related to hearing, such as otitis media and Meniere’s disease. The case history form provided a comprehensive baseline profile for each participant, aiding in the interpretation of audiological findings.

Supplementary file S1

### Audiological assessment

Audiological data were collected by qualified audiologists (first and third authors) using standardized procedures. Assessments were conducted in a sound-proof booth with the lowest ambient sound intensity to ensure accuracy. Testing adhered to protocols approved for clinical audiological practice in Saudi Arabia. The assessment involved pure-tone audiometry, which was conducted using calibrated audiometers to determine the air and bone conduction thresholds across octave and intra-octave frequencies ranging from 250 Hz to 8 kHz. The audiometric evaluations were performed using a single audiometer model and similar transducer (i.e., insert earphones) to ensure consistency. Ambient noise levels within the sound-proof booth met international standards (e.g., ANSI S3.1-1999).

## RESULTS

### Demographic and exposure characteristics

Table 1 illustrates the demographic and exposure characteristics of the total number of participants included in the current research. A total of 40 male participants were included in the study, with the majority aged 41–50 years (45%). Most participants (72.5%) reported serving in the military for 16 years or more, and 67.5% were currently employed. Prolonged exposure to loud noises (5 years or more) was prevalent among participants (77.5%), highlighting a substantial occupational risk factor. Regarding clinical symptoms, tinnitus was the most common complaint (60%), followed by both tinnitus and dizziness (12.5%). All participants had no hereditary hearing loss, and 70% reported no other medical conditions. Only ten participants (25%) reported health conditions, such as hypertension and diabetes mellitus. Additionally, all participants denied regular involvement in hobbies that involve high noise exposure. Bilateral hearing loss was more prevalent (87.5%) than unilateral (12.5%).

**Table 1:** Demographic information

Variables	Frequency *n* (%)
**Age (years)**	
18–30	3 (7.5)
31–40	13 (32.5)
41–50	18 (45)
51–60	6 (15)
**Military service duration (years)**	
1–4	2 (5)
5–9	4 (10)
10–15	5 (12.5)
≥ 16	29 (72.45)
**Currently employed**	
Yes	27 (67.5)
No	13 (32.5)
**Exposure to loud noise during military service (years)**	
< 1	2 (5)
1–2	4 (10)
3–4	3 (7.5)
≥ 5	31 (77.5)
**Symptoms of tinnitus and/or dizziness**	
Tinnitus	24 (60)
Tinnitus and dizziness	5 (12.5)
No	11 (27.5)
**Family history of hereditary hearing loss**	
Yes	0 (0)
No	40 (100)
**Other health conditions (e.g., diabetes, hypertension)**	
Yes	10 (25)
No	30 (75)
**Hobbies involving noise exposure**	
Yes	0 (0)
No	40 (100)
**Type of hearing loss**	
Unliteral (one ear)	4 (12.5)
Bilateral (Both ears)	34 (87.5)

### Audiometric profiles

**Figure 1:**
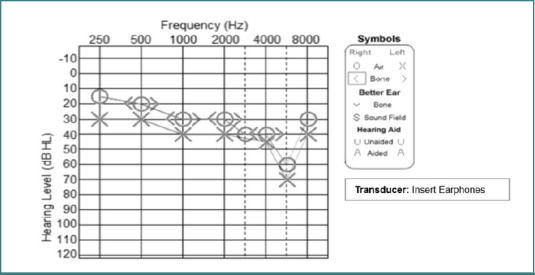
Audiogram showing air conduction hearing thresholds for both ears of a participant, measured using insert earphones. A characteristic noise-induced hearing loss notch is observed bilaterally at 6000 Hz, consistent with a history of noise exposure.

Table 2 shows the audiometric profiles of all the participants. Audiometric results revealed normal hearing thresholds sloping to mild, moderate, or severe SNHL in the right ear of 71.2% of participants and in the left ear of 53.0% of participants. Mild sloping to moderate, severe, or profound SNHL was more common in the left ear (21%) compared to the right (none). Moderate sloping to severe or profound SNHL were present in 15.3% of right ears, while mild and moderate SNHL were observed in the left ear at rates of 10.5% and 10.5%, respectively. Severe SNHL was present in 5% of left ears, but no severe cases were reported in the right ear. Figure 1 shows an example of one of the participants’ hearing thresholds.

**Table 2:** The number of affected ears with the severity of hearing loss

Affected ear	Severity of hearing loss^^^					
	Normal sloping to mild, moderate, or severe SNHL* *n* (%)	Mild sloping to moderate, severe, or profound SNHL* *n* (%)	Moderate sloping to severe or profound SNHL* *n* (%)	Mild SNHL* *n* (%)	Moderate SNHL* *n* (%)	Severe SNHL* *n* (%)
Right	27 (71.2)	0 (0)	6 (15.3)	3 (8.4)	2 (5.1)	0 (0)
Left	20 (53)	8 (21)	4 (10.5)	4 (10.5)	0 (0)	2 (5)

^Severity of hearing loss is based on hearing assessments. *SNHL, Sensorineural hearing loss.

### Associations between noise exposure and hearing loss

Table 3 depicts the association between noise exposure duration and audiometric outcomes. The majority of participants diagnosed with hearing loss experienced exposure for more than 2 years. Among those with more than 2 years of experience, 20.6% were diagnosed with mild sloping to moderate, severe, or profound SNHL, while 14.7% were diagnosed with mild SNHL. Additionally, 8.8% and 5.9% of participants experienced moderate and severe hearing loss, respectively. There was no statistical association between hearing loss and exposure to loud noises (*P* = 0.60). Most participants with more than 2 years of experience reported tinnitus and dizziness at rates of 61.8% and 14.7%, respectively, with no statistical association between symptoms (i.e., tinnitus and dizziness) and exposure to loud noises (*P* = 0.40).

**Table 3:** Association between the severity of hearing loss and symptoms with the period of loud noise exposure

Variables		Period of Loud Noise Exposure		*P* value
		Less than 2 years *n* (%)	More than 2 years *n* (%)	
Severity of hearing loss^	Normal sloping to mild, moderate, or severe SNHL*	5 (83.3)	13 (38.2)	0.60
	Mild sloping to moderate, severe, or profound SNHL*	0	7 (20.6)	
	Moderate sloping to severe or profound SNHL*	0	4 (11.8)	
	Mild SNHL*	1 (16.7)	5 (14.7)	
	Moderate SNHL*	0	3 (8.8)	
	Severe SNHL*	0	2 (5.9)	
Symptoms	Tinnitus	3 (50)	21 (61.8)	0.40
	Tinnitus and dizziness	0	5 (14.7)	
	No	3 (50)	8 (23.5)	

^Severity of hearing loss is based on hearing assessments. *SNHL, Sensorineural hearing loss.

## DISCUSSION

The current study provided critical insights into the risk factors and audiometric outcomes associated with NIHL among Saudi military personnel. The findings align with global evidence on the adverse effects of prolonged noise exposure in occupational settings, particularly in military environments characterized by high-intensity noise. Findings from this study also corroborate regional data [2,3]. Our study included only male participants, as there were limited female military personnel in Saudi Arabia during the study period, a finding consistent with other studies [6]. The observed predominance of bilateral SNHL and the correlation between prolonged noise exposure and worsening auditory thresholds are consistent with previous research. For instance, a study conducted among Saudi military personnel in Al Ahsa reported a high prevalence of hearing impairment (71.6%), which was attributed to prolonged exposure to hazardous noise and the limited use of hearing protection devices [6].

Bilateral hearing loss was predominant, aligning with previous studies on military populations exposed to occupational noise hazards [10]. Although the results lacked statistical significance, prolonged exposure to loud noises (over 2 years) correlated with a higher prevalence of severe SNHL. This aligns with evidence indicating cumulative auditory damage from chronic noise exposure [11]. The absence of severe SNHL in the short-exposure group highlights the potential protective effect of limited duration. Similarly, the higher prevalence of left-ear impairment was because it is positioned closer to the firearm's barrel, while the right ear is shielded by the head as it rests against the shoulder, creating an acoustic shadow for right-handed shooters [12].

Tinnitus, the most commonly reported symptom (60%), further highlights the impact of occupational noise exposure. Tinnitus often accompanies NIHL due to damage to cochlear hair cells [13]. While the association between prolonged exposure and tinnitus was not statistically significant, the trend warrants further investigation into individual susceptibility and co-factors. The low prevalence of severe SNHL among participants exposed for less than 2 years underscores the protective effect of limited exposure duration, reinforcing the WHO recommendations for noise exposure limits [11]. However, the lack of statistical significance in the results suggests that additional variables, such as the use of hearing protection devices, need to be explored. Despite a high prevalence of tinnitus and dizziness symptoms, statistical significance was not achieved, suggesting potential variability in individual susceptibility [13]. Interestingly, medical comorbidities did not emerge as major contributors in this cohort, suggesting occupational noise as the primary etiological factor. Implementing comprehensive conservation programs that include regular auditory assessments, the mandatory use of hearing protection devices, and awareness campaigns is essential to mitigate the risk of NIHL. This aligns with a local study that identified a lack of systematic training and enforcement of hearing protection protocols among Saudi industrial workers [14].

### Study limitations and future research

This study has a few limitations. Although this is a preliminary study that serves as a pilot study before a full-scale research project, the small sample size may be considered a limitation. Future research with larger cohorts and objective measures of noise exposure could strengthen the evidence base. Additionally, the lack of statistical significance in the associations underscores the need for further exploration of confounding factors, such as the use of hearing protection and the type of military weapon used. The lack of control for confounders reported by a few participants, such as noisy hobbies, was another limitation. All the above-mentioned limitations should be eliminated to refine research methods for a larger study.

## CONCLUSION

Prolonged noise exposure remains a critical risk factor for bilateral SNHL among military personnel. Our study revealed several audiometric findings and different SNHL profiles among the participants, particularly those with extended exposure durations. Bilateral SNHL and tinnitus were more prevalent. Factors that might contribute to this NIHL include limited awareness of NIHL, inadequate training on the use of hearing protection devices, and the absence of stringent enforcement of protective protocols. This study was also helpful in examining the study protocol and evaluating the acceptability and feasibility of a future larger study. The need for comprehensive hearing conservation programs tailored to the military context is evident in Saudi Arabia. Such programs should encompass regular auditory assessments, mandatory use of appropriate hearing protection, and educational initiatives to raise awareness about the risks of noise exposure and the importance of preventive measures.

## Data Availability

Further data is available from the corresponding author upon reasonable request.
