# Supplementary material for: Noise-induced hearing loss among military personnel in Saudi Arabia: a preliminary study
Source: J Med Life. 2025 Jun;18(6):552–6. doi: 10.25122/jml-2025-0013 (PMC12314848; doi:10.25122/jml-2025-0013)
Supplement: Supplementary file 1 [file JMedLife-18-552-s001.pdf]

1. Gender

- ☐ Male
- ☐ Female

2. What is your age? (Years)

- ☐ 18–30
- ☐ 31–40
- ☐ 41–50
- ☐ 51–60

3. How long did you serve in the military? (Years)

- ☐ 1–4
- ☐ 5–9
- ☐ 10–15
- ☐ >16

4. Are you currently employed?

- ☐ Yes
- ☐ No

5. How long have you been or have you been exposed to loud noises during military services? (Years)

- ☐ < 1
- ☐ 1–2
- ☐ 3–4
- ☐ >5

6. Do you suffer, or have you suffered from symptoms of tinnitus and/or dizziness?

- ☐ Tinnitus
- ☐ Tinnitus and dizziness
- ☐ No

7. Is there a history of hereditary hearing loss in your family?

- ☐ Yes
- ☐ No

8. Do you suffer from any other health conditions (e.g., diabetes mellitus, hypertension, etc.)?

- ☐ Yes
- ☐ No

9. Do you currently engage in, or have you previously engaged in, hobbies that involve exposure to loud noises? (e.g., riding motorcycles, hunting with guns, listening to loud music, etc.)

- ☐ Yes
- ☐ No

10. What is the nature of your hearing loss?

- ☐ Unliteral (one ear)
- ☐ Bilateral (Both ears)

© 2025 by the authors. This article is an open access article distributed under the terms and conditions of the Creative Commons Attribution (CC BY 4.0) license.
